# Clinical performance and health equity implications of the American Diabetes Association’s 2023 screening recommendation for prediabetes and diabetes

**DOI:** 10.3389/fendo.2023.1279348

**Published:** 2023-10-13

**Authors:** Matthew J. O’Brien, Yan Zhang, Stacy C. Bailey, Sadiya S. Khan, Ronald T. Ackermann, Mohammed K. Ali, Michael E. Bowen, Stephen R. Benoit, Giuseppina Imperatore, Christopher S. Holliday, Kai McKeever Bullard

**Affiliations:** ^1^ Division of General Internal Medicine and Geriatrics, Department of Medicine, Northwestern University Feinberg School of Medicine, Chicago, IL, United States; ^2^ Institute for Public Health and Medicine, Northwestern University Feinberg School of Medicine, Chicago, IL, United States; ^3^ Chicago Center for Diabetes Translation Research, Northwestern University Feinberg School of Medicine and University of Chicago Pritzker School of Medicine, Chicago, IL, United States; ^4^ Department of Preventive Medicine, Northwestern University Feinberg School of Medicine, Chicago, IL, United States; ^5^ Division of Diabetes Translation, National Center for Chronic Disease Prevention and Health Promotion, Centers for Disease Control and Prevention, Atlanta, GA, United States; ^6^ Division of Cardiology, Department of Medicine, Northwestern University Feinberg School of Medicine, Chicago, IL, United States; ^7^ Hubert Department of Global Health, Rollins School of Public Health, Emory University, Atlanta, GA, United States; ^8^ Department of Family and Preventive Medicine, School of Medicine, Emory University, Atlanta, GA, United States; ^9^ Division of General Internal Medicine, Department of Medicine, University of Texas Southwestern Medical Center, Dallas, TX, United States; ^10^ Peter O’Donnell Jr. School of Public Health, University of Texas Southwestern Medical Center, Dallas, TX, United States

**Keywords:** diabetes screening, prediabetes screening, health equity, racial and ethnic disparities, sex and gender disparities, population health

## Abstract

**Introduction:**

The American Diabetes Association (ADA) recommends screening for prediabetes and diabetes (dysglycemia) starting at age 35, or younger than 35 years among adults with overweight or obesity and other risk factors. Diabetes risk differs by sex, race, and ethnicity, but performance of the recommendation in these sociodemographic subgroups is unknown.

**Methods:**

Nationally representative data from the National Health and Nutrition Examination Surveys (2015-March 2020) were analyzed from 5,287 nonpregnant US adults without diagnosed diabetes. Screening eligibility was based on age, measured body mass index, and the presence of diabetes risk factors. Dysglycemia was defined by fasting plasma glucose ≥100mg/dL (≥5.6 mmol/L) or haemoglobin A1c ≥5.7% (≥39mmol/mol). The sensitivity, specificity, and predictive values of the ADA screening criteria were examined by sex, race, and ethnicity.

**Results:**

An estimated 83.1% (95% CI=81.2-84.7) of US adults were eligible for screening according to the 2023 ADA recommendation. Overall, ADA’s screening criteria exhibited high sensitivity [95.0% (95% CI=92.7-96.6)] and low specificity [27.1% (95% CI=24.5-29.9)], which did not differ by race or ethnicity. Sensitivity was higher among women [97.8% (95% CI=96.6-98.6)] than men [92.4% (95% CI=88.3-95.1)]. Racial and ethnic differences in sensitivity and specificity among men were statistically significant (*P*=0.04 and *P*=0.02, respectively). Among women, guideline performance did not differ by race and ethnicity.

**Discussion:**

The ADA screening criteria exhibited high sensitivity for all groups and was marginally higher in women than men. Racial and ethnic differences in guideline performance among men were small and unlikely to have a significant impact on health equity. Future research could examine adoption of this recommendation in practice and examine its effects on treatment and clinical outcomes by sex, race, and ethnicity.

## 1 Introduction

Diabetes affects 529 million people worldwide, which is projected to increase to more than 1.31 billion by 2050 (1). Over half of US adults have prediabetes or type 2 diabetes (collectively called dysglycemia) (2, 3), significantly impacting the health and quality of life among those affected and accounting for over $400 billion in annual healthcare spending (4). Approximately one-quarter of US adults with diabetes are undiagnosed and more than 80% of those with prediabetes are unaware of having the condition (2, 3). The importance of screening for dysglycemia is supported by these data, as well as the availability of evidence-based treatments to prevent and manage type 2 diabetes.

The American Diabetes Association (ADA) currently recommends starting dysglycemia screening at age 35 years, or among adults younger than 35 years who have overweight or obesity and at least one other diabetes risk factor (5). Starting in 2022, ADA lowered the recommended screening age from the prior threshold of 45 years (6). While lowering the age cutoff for the entire US adult population expands screening eligibility and increases the proportion of those with dysglycemia who are eligible (7), it is not known how this age change impacts performance of the current screening criteria in key demographic subgroups.

A large body of evidence demonstrates that individuals from racial and ethnic minority groups develop diabetes at younger ages than non-Hispanic White (hereafter, White) adults. For example, one recent analysis found that non-Hispanic Black (hereafter, Black), non-Hispanic Asian (hereafter, Asian), and Hispanic adults were almost twice as likely as White adults to be diagnosed with diabetes before age 35 (8). These data suggest that ADA’s current screening recommendation may promote health equity by identifying a greater proportion of young adults from racial and ethnic minority groups who have dysglycemia. Because the guideline also recommends screening young adults before age 35 if they have overweight or obesity and other diabetes risk factors, the higher prevalence of these related conditions among young adults from racial and ethnic minority groups may result in even greater screening eligibility among those with the highest diabetes risk (9–11).

Prior research also documents sex differences in the burden of diabetes and related risk factors. Overall, men have a marginally higher prevalence of diabetes (12.6%) than women (10.2%) (3). However, women are at higher risk of developing diabetes at younger ages, with men exhibiting higher risk during middle age, and both sexes experiencing similar diabetes risk as older adults (12). Women have a higher prevalence of obesity than men (13), and some experience risk factors like gestational diabetes that are unique to women. These sex-based differences in diabetes risk may impact the performance of ADA’s current screening recommendation.

The objective of this study was to examine performance of the 2023 ADA screening criteria by sex, race, and ethnicity. Using nationally representative data, we describe eligibility according to the current ADA guideline, as well as its clinical performance characteristics, across relevant sociodemographic groups.

## 2 Materials and methods

### 2.1 Study data and participants

We analyzed data from the National Health and Nutrition Examination Surveys (NHANES) from 2015 to March 2020. NHANES conducts interviews and collects biological specimens describing the health of the non-institutionalized US civilian population, using a complex, multistage probability sample design. Protocols for collecting NHANES data have been published elsewhere (14). All participants completed an in-person examination with measurement of height and weight to calculate body mass index (BMI), and a blood sample measuring haemoglobin A1c (A1c). Approximately half of participants also completed a fasting blood sample that included fasting plasma glucose (FPG). The study sample comprised adults aged ≥18 years who underwent fasting blood collection and did not have a self-reported diagnosis of diabetes. We excluded pregnant people and those who were missing data for self-report of clinician-diagnosed diabetes, BMI, or glycaemic measures (n=9). The total analytic sample included 5,287 participants. NHANES was approved by the Ethics Review Board of the National Center for Health Statistics. Each adult participant provided written consent.

### 2.2 Key variables

Screening eligibility according to the 2023 ADA recommendation was based on age ≥35 years. The presence of overweight or obesity (defined by BMI ≥23kg/m^2^ in Asian Americans and BMI ≥25kg/m^2^ in all other groups) and at least one of the following diabetes risk factors were used to determine screening eligibility among adults aged 18-34 years: minority race or ethnicity; hypertension; dyslipidaemia; history of prediabetes; family history of diabetes; history of cardiovascular disease; history of gestational diabetes; and physical inactivity. Definitions of these diabetes risk factors using NHANES variables are listed in **Supplementary Table 1**.

NHANES includes self-reported data for race and ethnicity, including Asian (comprising those with origins in the Far East, Southeast Asia, or the Indian subcontinent), Black, Hispanic, and White adults. Participants reporting the following races were not included due to small sample sizes: American Indian, Alaska Native, Native Hawaiian and Pacific Islander. Sex is also based on participants’ self-report. According to ADA practice standards, prediabetes was defined by FPG 100-125 mg/dL (5.6-6.9 mmol/L) or A1c 5.7-6.4% (39-46 mmol/mol), and diabetes was defined by FPG ≥126 mg/dL (≥7.0 mmol/L) or A1c ≥6.5% (≥48 mmol/mol) (5). We also analyzed data on the following participant characteristics that are associated with diabetes risk: waist circumference (i.e., measured at the level of the iliac crest), educational attainment (i.e., the highest level of education completed), household income (i.e., the total combined income of all household members), insurance status (i.e., health insurance coverage), and having a usual source of care (i.e., a place to go when sick or need advice about health) (5, 15).

### 2.3 Statistical analysis

We used descriptive statistics to characterize US adults who were eligible for screening according to the 2023 ADA screening recommendation. The characteristics of participants with prediabetes and undiagnosed diabetes were also assessed using descriptive statistics, defining the population that screening efforts are intended to detect. These descriptive analyses were stratified by sex, as well as race and ethnicity. In the full sample, the following performance characteristics of the ADA screening criteria were calculated: sensitivity (the proportion of adults with dysglycemia who meet the screening criteria); specificity (the proportion of those without dysglycemia who do not meet the screening criteria); positive predictive value (the proportion of adults meeting the screening criteria who have dysglycemia); and negative predictive value (the proportion of those not meeting the screening criteria who are free of dysglycemia). The significance of differences between all subgroups was assessed using chi-square tests for racial and ethnic categories. Sensitivity analyses examined performance characteristics of the ADA screening criteria using FPG alone and A1c alone to define prediabetes and diabetes. SAS-callable SUDAAN, version 9.4 was used to conduct statistical analyses using fasting sample weights (SAS Institute, Cary, NC; RTI International, Research Triangle Park, NC). We estimated 95% confidence intervals using PROC RLOGIST and considered a p-value of <0.05 to be statistically significant for all analyses.

## 3 Results

An estimated 83.1% (95% CI=81.2-84.7) of US adults without diagnosed diabetes were eligible for dysglycemia screening according to the 2023 ADA criteria, representing 179 million adults (**Table 1**). In general, eligible men were marginally younger than eligible women, and the mean age of eligible adults was significantly higher for White participants than those from other racial and ethnic groups. Except for Asian adults, the mean BMI was lower among eligible men than women, with sex-based differences that were significant for Black participants. Mean FPG levels were similar across racial and ethnic groups, with eligible Hispanic and White men having higher values than their female counterparts. Mean A1c values were similar across all subgroups defined by sex, race, and ethnicity. The prevalence of diabetes risk factors was also similar across all demographic groups. Men were less likely to report having a usual source of care than women in all racial and ethnic groups.

**Table 1:** Characteristics of U.S. adults eligible for prediabetes and diabetes screening according to ADA criteria by self-reported sex, race, and ethnicity.

Characteristic	Asian		Black		Hispanic		White	
	Men	Women	Men	Women	Men	Women	Men	Women
	% (95%CI)	% (95%CI)	% (95%CI)	% (95%CI)	% (95%CI)	% (95%CI)	% (95%CI)	% (95%CI)
Unweighted n	283	300	486	563	558	637	749	788
Mean age, years	43.0 (41.1-45.0)	48.0 (45.8-50.1)	46.1 (44.1-48.1)	46.2 (44.6-47.8)	40.7 (38.9-42.5)	44.0 (42.1-45.8)	51.9 (50.8-53.1)	54.6 (53.7-55.4)
Age categories, years								
18-34	35.0 (28.8-41.8)	19.9 (15.5-25.2)	25.7 (20.7-31.6)	28.7 (24.7-33.1)	35.6 (30.8-40.7)	29.0 (24.6-33.8)	13.7 (11.0-17.0)	11.0 (8.7-13.9)
35-44	23.0 (16.8-30.6)	24.3 (19.9-29.4)	23.5 (18.0-30.1)	17.4 (13.9-21.4)	27.4 (23.0-32.3)	25.8 (21.6-30.5)	18.9 (15.8-22.4)	15.7 (13.4-18.2)
45-70	37.2 (31.0-43.9)	49.3 (42.6-56.0)	44.3 (37.7-51.1)	46.2 (42.2-50.3)	33.2 (28.8-37.9)	39.4 (35.5-43.5)	55.8 (51.4-60.2)	56.1 (52.0-60.2)
≥71	4.7 (2.6-8.4)	6.6 (4.1-10.3)	6.5 (4.5-9.1)	7.7 (5.4-10.8)	3.8 (2.2-6.5)	5.8 (3.8-8.7)	11.5 (9.3-14.2)	17.2 (14.7-20.0)
Education < high school	12.4 (9.0-16.8)	14.3 (10.7-18.9)	13.2 (10.1-17.1)	11.4 (8.4-15.2)	36.3 (31.2-41.6)	31.0 (25.2-37.4)	8.8 (6.4-11.9)	6.8 (5.0-9.2)
Income below Federal Poverty Level	8.9 (5.7-13.5)	8.4 (5.6-12.4)	22.2 (17.7-27.5)	27.9 (22.0-34.6)	28.8 (23.2-35.2)	31.2 (24.4-38.9)	6.0 (4.0-8.7)	8.2 (6.1-11.0)
Insured	92.7 (84.3-96.7)	94.0 (89.8-96.5)	76.6 (70.7-81.7)	85.8 (80.2-90.0)	61.4 (51.8-70.2)	73.7 (66.4-79.9)	89.5 (85.0-92.8)	93.4 (91.0-95.1)
Usual source of care	73.6 (65.4-80.4)	83.9 (78.0-88.5)	74.6 (67.5-80.6)	90.0 (84.3-93.8)	62.4 (54.4-69.7)	82.4 (74.3-88.4)	82.1 (76.0-86.9)	90.1 (85.2-93.5)
Weight status^a^								
Normal	14.5 (10.6-19.6)	27.8 (20.7-36.2)	18.0 (13.7-23.2)	8.9 (6.6-11.8)	6.8 (4.7-9.7)	12.7 (10.1-16.0)	17.8 (14.6-21.5)	26.2 (22.2-30.6)
Overweight	71.8 (64.2-78.3)	56.3 (48.0-64.2)	41.5 (36.5-46.7)	27.9 (23.4-32.9)	47.5 (42.7-52.4)	34.6 (31.1-38.2)	40.3 (36.1-44.6)	32.0 (28.1-36.1)
Obesity	13.7 (10.2-18.2)	15.9 (11.6-21.5)	40.5 (36.0-45.2)	63.2 (57.9-68.3)	45.7 (41.2-50.3)	52.7 (47.8-57.6)	41.9 (36.7-47.3)	41.9 (37.9-45.9)
Body mass index, kg/m^2 b^	26.4 (25.9-27.0)	25.8 (25.1-26.5)	29.9 (29.2-30.6)	34.0 (33.1-34.8)	30.7 (30.2-31.2)	31.5 (30.7-32.3)	29.8 (29.1-30.4)	30.2 (29.4-30.9)
Hypertension	44.2 (38.5-50.1)	40.4 (33.6-47.5)	59.1 (52.5-65.4)	58.2 (52.3-64.0)	40.0 (35.4-44.9)	31.2 (27.6-35.0)	55.5 (51.0-59.9)	47.8 (43.3-52.4)
Dyslipidaemia	25.3 (19.4-32.3)	16.4 (12.5-21.1)	18.9 (14.9-23.7)	16.8 (14.0-20.0)	22.4 (18.9-26.3)	14.2 (11.3-17.7)	33.6 (29.5-38.0)	24.3 (20.3-28.9)
Self-reported prediabetes	12.4 (8.4-17.9)	21.1 (14.4-29.9)	14.4 (11.7-17.6)	18.3 (15.7-21.3)	14.1 (11.3-17.3)	19.1 (16.2-22.4)	13.3 (9.8-17.8)	16.1 (13.4-19.3)
Family history of diabetes	29.1 (24.0-34.8)	44.9 (38.3-51.6)	41.1 (35.5-46.9)	52.1 (45.4-58.7)	37.3 (31.9-43.0)	50.3 (45.0-55.7)	39.8 (36.0-43.7)	44.5 (39.6-49.4)
History of gestational diabetes^c^	–	11.7 (8.2-16.3)	–	11.5 (9.1-14.5)	–	19.0 (15.9-22.5)	–	19.5 (16.1-23.4)
History of cardiovascular disease	3.0 (1.5-6.0)	0.9 (0.2-3.3)	7.3 (5.1-10.3)	7.6 (5.8-10.0)	3.2 (1.9-5.5)	3.4 (2.5-4.7)	9.1 (6.7-12.2)	7.7 (5.0-11.6)
Physical inactivity	16.1 (12.2-20.9)	30.5 (24.2-37.6)	21.0 (17.3-25.2)	29.2 (23.0-36.3)	18.0 (14.6-22.1)	31.2 (26.6-36.3)	12.1 (9.5-15.4)	23.5 (19.6-27.9)
Received glucose test in last 3 years	51.3 (44.6-58.0)	56.1 (49.8-62.3)	49.2 (42.2-56.3)	61.1 (56.7-65.3)	43.8 (37.6-50.1)	58.0 (53.3-62.7)	55.1 (51.4-58.6)	55.6 (51.3-59.7)
Fasting plasma glucose, mg/dL^b^	106.7 (103.4-109.9)	103.2 (100.2-106.2)	102.1 (100.6-103.6)	102.1 (100.0-104.2)	106.9 (105.2-108.6)	103.6 (101.3-106.0)	106.6 (105.1-108.0)	102.4 (101.1-103.8)
100-125	35.4 (28.3-43.3)	30.7 (24.0-38.3)	28.6 (23.3-34.5)	27.2 (21.9-33.3)	42.5 (37.0-48.3)	30.1 (26.5-34.0)	48.5 (42.7-54.3)	31.1 (27.2-35.4)
≥126	5.1 (3.0-8.5)	3.9 (1.8-8.4)	2.6 (1.5-4.5)	2.7 (1.7-4.3)	4.2 (2.9-6.1)	4.3 (2.6-7.0)	2.8 (1.8-4.4)	2.7 (1.5-4.6)
Hemoglobin A1c, %^b^	5.59 (5.50-5.69)	5.63 (5.52-5.73)	5.59 (5.54-5.65)	5.68 (5.61-5.74)	5.55 (5.48-5.61)	5.56 (5.50-5.62)	5.47 (5.43-5.50)	5.50 (5.46-5.54)
5.7-6.4	30.1 (24.7-36.0)	33.2 (26.7-40.5)	41.9 (37.2-46.7)	42.2 (37.5-47.0)	26.0 (23.1-29.1)	28.0 (24.5-31.8)	26.6 (22.2-31.4)	28.2 (23.4-33.5)
≥6.5	4.2 (2.2-7.9)	5.4 (2.9-9.9)	3.9 (2.4-6.4)	4.9 (3.4-6.9)	4.3 (3.2-5.9)	4.0 (2.5-6.4)	1.3 (0.7-2.3)	2.7 (1.8-4.2)

^a^According to the 2023 ADA guideline, weight status was defined in Asian adults using the following BMI thresholds: Normal (18.0-22.9), Overweight (23.0-26.9), and Obesity (≥27.0). In all other racial and ethnic groups, weight status was defined using the following BMI thresholds: Normal (18.0-24.9), Overweight (25.0-29.9), and Obesity (≥30.0).

^b^Values are reported as mean (standard error).

^c^History of gestational diabetes was only assessed among those reporting female sex.

**Figure 1:**
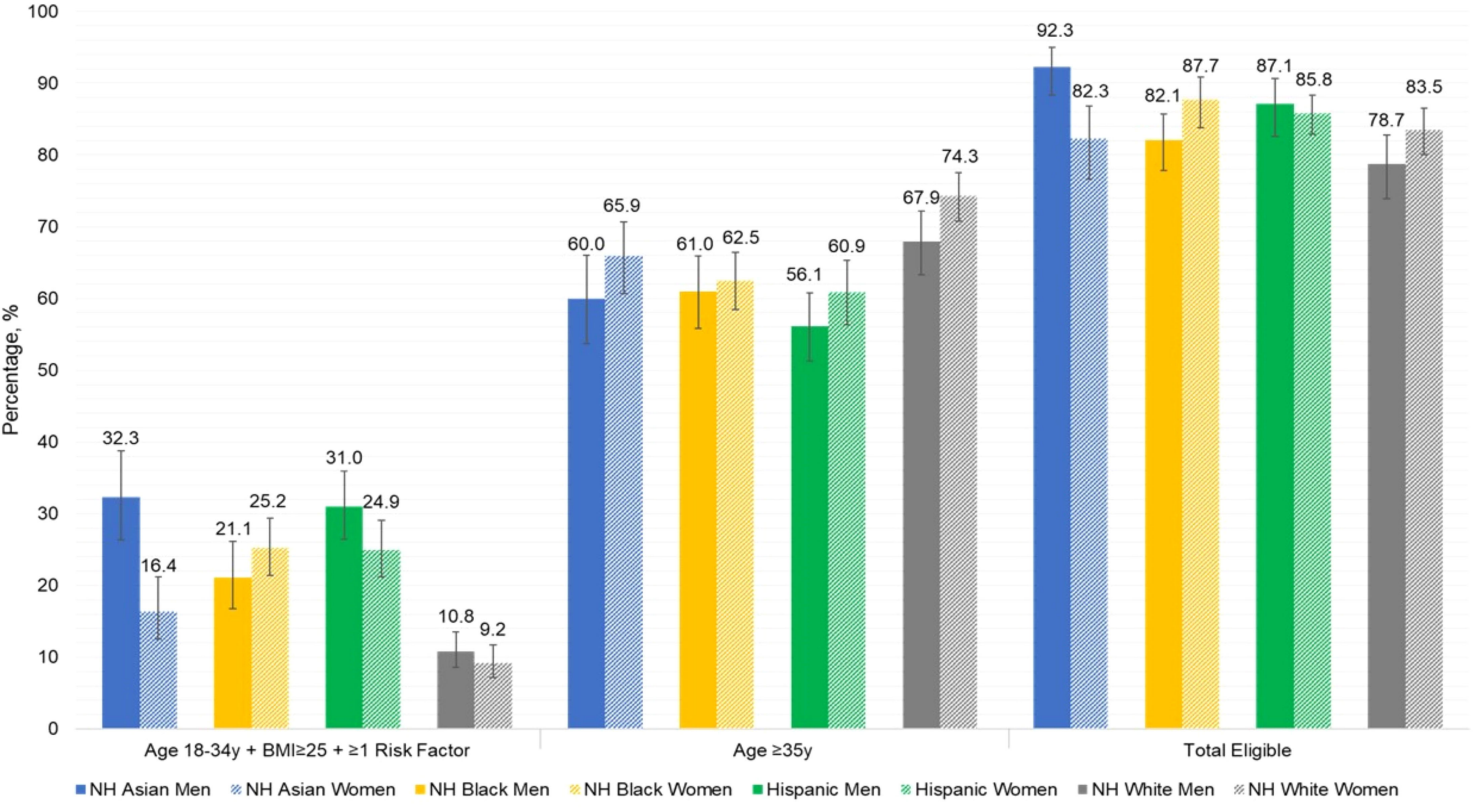
Proportion of U.S. adults eligible for dysglycemia screening according to current ADA criteria, by sex, race, and ethnicity.

**Figure 2:**
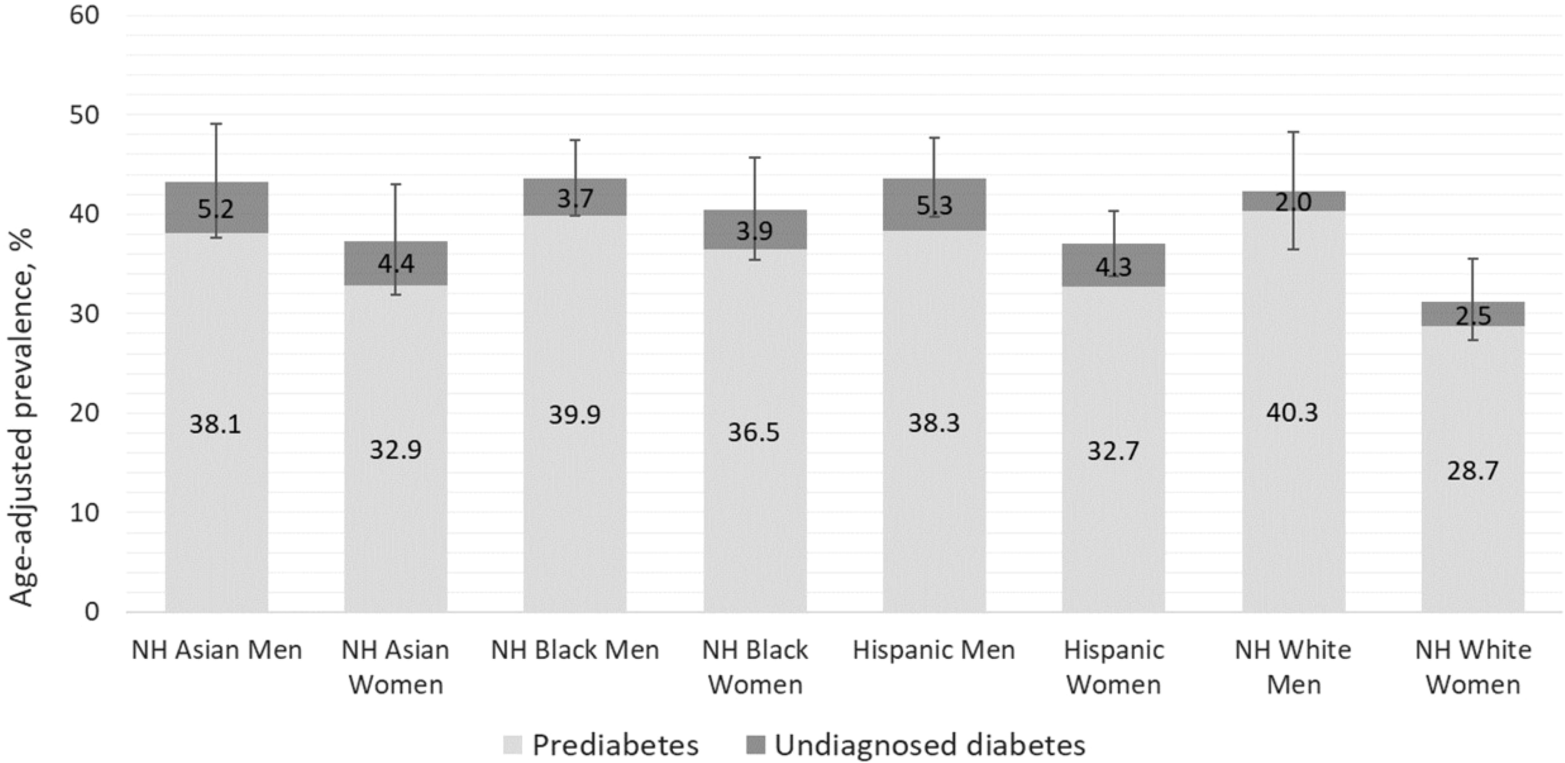
Prevalence of prediabetes and undiagnosed diabetes among U.S. adults by sex, race, and ethnicity.

**Figure 1** presents the proportion of US adults who are eligible for dysglycemia screening according to age ≥35 years alone, age 18-34 years with overweight or obesity and additional diabetes risk factors, and the total eligible population, with stratification by sex, race, and ethnicity. More adults were eligible for screening based on age ≥35 years, compared to those younger than age 35 with diabetes risk factors. Asian men were most likely to be eligible, and differences in screening eligibility across demographic subgroups were small. **Figure 2** displays the combined prevalence of prediabetes and undiagnosed diabetes, which was marginally higher among men within each racial and ethnic group. White women were significantly less likely to have dysglycemia than other demographic groups.

**Supplementary Table S2** displays the characteristics of US adults with prediabetes or undiagnosed diabetes, whom screening efforts are meant to identify, with stratification by sex, race, and ethnicity.

**Table 2** displays the clinical performance characteristics of the 2023 ADA screening criteria by sex, race, and ethnicity. In the entire adult population, sensitivity was very high, and specificity was low, with higher negative predictive value than positive predictive value. The screening criteria exhibited higher sensitivity among women [97.8% (95% CI: 96.6-98.6)] than men [92.4% (95% CI: 88.3-95.1)]. There was greater variation in sensitivity observed among men, ranging from 89.6% (95% CI: 83.0-93.9) in Black men to 97.9% (95% CI: 94.7-99.1) in Asian men (*P*=0.04). Specificity of the ADA screening criteria also differed significantly among men from different racial and ethnic groups (*P*=0.02), with the highest specificity observed among White men and the lowest found among Asian men [34.4%, (95% CI 27.9-41.4) *vs*. 12.9%, (95% CI: 7.9-20.3)]. In women, the screening criteria exhibited lower specificity than men overall, as well as less variation by race and ethnicity (*P*=0.61).

**Table 2:** Performance of ADA screening criteria among U.S. adults without diagnosed diabetes by self-reported sex, race, and ethnicity.

Population group	Sensitivity (95% CI)	Specificity (95% CI)	Positive Predictive Value (95% CI)	Negative Predictive Value (95% CI)
Total population				
Overall	95.0 (92.7-96.6)	27.1 (24.5-29.9)	52.5 (49.5-55.5)	86.3 (80.1-90.8)
Asian	98.0 (95.5-99.1)	22.1 (16.9-28.3)	51.1 (44.9-57.3)	93.0 (85.6-96.7)
Black	94.3 (91.1-96.4)	23.5 (18.9-28.9)	54.5 (49.4-59.4)	81.1 (73.5-86.9)
Hispanic	95.5 (92.9-97.2)	21.1 (17.1-25.6)	50.0 (46.4-53.5)	85.0 (78.2-89.9)
White	94.6 (90.5-96.9)	29.9 (25.8-34.3)	52.8 (48.4-57.2)	86.9 (77.1-92.9)
*P*-value^a^	0.12	0.14	0.43	0.13
Men				
Overall	92.4 (88.3-95.1)	29.3 (25.2-33.7)	56.9 (52.5-61.1)	79.2 (69.5-86.4)
Asian	97.9 (94.7-99.1)	12.9 (7.9-20.3)	51.3 (44.2-58.3)	86.5 (71.9-94.1)
Black	89.6 (83.0-93.9)	24.9 (18.7-32.4)	54.5 (48.5-60.3)	70.5 (57.2-81.1)
Hispanic	94.7 (90.6-97.1)	20.2 (14.2-27.9)	53.3 (47.8-58.7)	79.8 (67.9-88.1)
White	91.4 (84.4-95.4)	34.4 (27.9-41.4)	59.2 (52.8-65.3)	79.4 (65.3-88.7)
*P*-value^a^	0.04	0.02	0.18	0.35
Women				
Overall	97.8 (96.6-98.6)	25.4 (22.3-28.7)	48.6 (45.5-51.7)	94.1 (90.6-96.4)
Asian	98.1 (94.1-99.4)	29.5 (21.5-39.0)	50.9 (42.6-59.1)	95.5 (86.5-98.6)
Black	98.3 (97.0-99.1)	22.5 (16.8-29.4)	54.4 (48.3-60.4)	93.5 (88.3-96.5)
Hispanic	96.5 (92.6-98.4)	21.8 (17.7-26.5)	46.5 (41.9-51.2)	89.8 (79.8-95.2)
White	98.2 (96.8-99.0)	26.5 (22.0-31.6)	47.3 (42.5-52.1)	95.7 (91.6-97.9)
*P*-value^a^	0.68	0.61	0.07	0.49

ADA, American Diabetes Association; CI, confidence interval.

^a^P-values for sociodemographic differences in performance characteristics were determined using chi-square tests.

Positive predictive value of the ADA screening criteria was significantly higher in men than women. There were relatively small racial and ethnic differences in positive predictive value by race and ethnicity in both women and men, which were not statistically significant. The negative predictive value among all women was 94.1% (95% CI: 90.6-96.4), which was significantly higher than among men [79.2% (95% CI: 69.5-86.4)]. Observed variability in negative predictive values by race and ethnicity were not statistically significant (*P*=0.49 in women and *P*=0.35 in men).

Sensitivity analyses demonstrated similar performance of the ADA screening criteria when defining dysglycemia based on FPG alone and A1c alone (**Supplementary Tables S3**, **S4**). Using A1c, small differences in performance characteristics by race and ethnicity were statistically significant. Using FPG, there were fewer statistically significant differences in performance by sex, race, and ethnicity. Sensitivity in Black men was similar to other groups when using FPG alone; and sensitivity among White men was comparable with other groups when using A1c alone. Less variation in performance of the screening criteria was observed among women based on FPG or A1c alone.

## 4 Discussion

This is the first study examining health equity implications of the current ADA dysglycemia screening recommendation. Overall, 83.1% of US adults without diabetes are eligible for screening according to the ADA criteria, most of whom were eligible based on age ≥35 years. Adults from racial and ethnic minority groups were eligible at younger ages than White adults, enhancing the potential to detect prediabetes and diabetes early among those at the highest risk. In the entire US adult population, the ADA screening criteria exhibited very high sensitivity and negative predictive value. These performance characteristics were marginally higher in women than men. Specificity was low across the US adult population, with significant differences observed by race and ethnicity in men. Variation in the ADA recommendation’s performance by sex, race, and ethnicity was generally small and unlikely to have a significant impact on health equity. Our finding that approximately half of US adults reported prior glucose testing suggests that achieving health equity may depend more on effective implementation of the 2023 ADA screening criteria across all sociodemographic groups.

Our study examined performance of the current ADA screening recommendation, which is the most widely followed dysglycemia screening guideline (16). Health equity implications of the United States Preventive Services Task Force screening guideline were examined previously, highlighting lower sensitivity and higher specificity than the ADA screening criteria (17). Because the ADA recommendation maximizes sensitivity at the expense of lower specificity, it has greater potential to promote health equity in detecting prediabetes and diabetes, while likely increasing healthcare costs by testing more adults unaffected by these conditions.

Determining the best approach for dysglycemia screening requires evaluating trade-offs in the clinical performance characteristics examined here. Because the ADA recommendation maximizes sensitivity, estimated at 94.9% across the entire US adult population (7), our findings indicate that the overwhelming majority of those with prediabetes and undiagnosed diabetes will be eligible for screening. The negative predictive value of the ADA screening recommendation was similarly high, suggesting that most people who are not eligible are not likely to have dysglycemia and therefore should not be tested. Low specificity of the ADA screening criteria represents the primary trade-off, with a significant proportion of adults without dysglycemia still being eligible for testing.

The performance of screening recommendations should also be interpreted based on the target health condition, and the tests used to identify them. Having high sensitivity that maximizes dysglycemia detection is supported by the availability of intensive lifestyle interventions found to prevent diabetes among adults with prediabetes, and evidence-based diabetes treatments than can improve glycaemic control and prevent complications (18–20). However, following the ADA recommendation also means that many adults without dysglycemia would also be tested due to low specificity, increasing healthcare costs and exposing more individuals to the risks of screening. These could be considered minor concerns given the low cost of glycaemic tests, compared with more costly screening tests like mammography and colonoscopy, and the minimal risks associated with venipuncture (21). Choosing the optimal approach for dysglycemia screening requires balancing the urgency of identifying and treating this condition against the potential risks to patients and costs to the healthcare system, factors that could be weighed differently by diverse stakeholders.

The ADA screening criteria exhibit small differences in performance between men and women. Sensitivity was significantly higher in women, likely reflecting sex-based differences in body weight and composition. US women younger than age 40 are more likely to have obesity than their male counterparts (37.0% *vs*. 31.6%, respectively) (22), which may increase eligibility in young women given that the screening criteria require having overweight or obesity below age 35 years. Differences in body fat distribution may also affect differential screening eligibility and guideline performance by sex. Men have greater visceral adipose tissue than women at the same BMI (23), which is associated with a greater risk of developing dysglycemia (24). As a result, men exhibit lower BMIs at dysglycemia diagnosis and are more likely than women to have dysglycemia at a normal BMI (25, 26), when they are ineligible for screening below age 35 years.

The sex differences observed in performance characteristics may also be influenced by diabetes risk factors that are unique to women. Recent estimates indicate that approximately 6% of pregnancies are complicated by gestational diabetes (27), which should prompt dysglycemia screening according to the ADA recommendation. While the prevalence of this diabetes risk factors is low, its inclusion in the screening criteria may modestly increase sensitivity and negative predictive value in women.

The ADA screening criteria also exhibited small variation by race and ethnicity. Racial and ethnic differences in performance characteristics were not statistically different in the overall adult population and among women. Sensitivity was significantly lower among Black and White men than those from other racial and ethnic groups. We also found significant racial and ethnic variation in specificity among men, which was lowest in Asian men. Asian adults exhibit a higher percentage of body fat at any BMI relative to White adults, as well as higher levels of visceral fat accumulation (28). These differences in body composition and body fat distribution have informed expert recommendations to begin screening Asian adults for dysglycemia at BMIs ≥23kg/m^2^ (29). Despite using this lower BMI cutoff in our analysis, we still observed lower specificity in Asian men compared with other racial and ethnic groups.

Evaluating the potential impact of observed racial and ethnic differences in guideline performance requires considering how they may impact receipt of screening and downstream outcomes. Lower specificity of the ADA screening criteria in Asian men means that a greater proportion of those without dysglycemia are eligible. Therefore, following this recommendation will screen more Asian men without these conditions than men from other racial or ethnic groups. This approach may increase healthcare costs; however, it is unlikely to impact health equity. Because sensitivity of the ADA criteria is so high, following this recommendation will identify most adults with dysglycemia across racial and ethnic groups. Experts warn against using race-based clinical algorithms as a strategy for promoting health equity (30), given the scientific consensus that race is a social construct rather than a biologic one (31). We observed differential sensitivity in White and Black men using A1c *vs*. FPG, which suggests that completing both tests will increase detection of dysglycemia in these groups. The ADA recommendation includes both FPG and A1c, allowing clinicians flexibility to choose the test or tests they prefer for each patient.

As the first study to examine health equity implications of the current ADA screening recommendation, our analysis is timely and responds to a renewed imperative to reduce racial and ethnic diabetes disparities. This study is also the first to examine variation in guideline performance by sex, highlighting some differences that may maximize dysglycemia detection and minimize unnecessary screening among women. Our use of the most recent nationally representative data from NHANES represents another strength. Examining performance of the ADA screening criteria separately by A1c and FPG is novel, while noting small differences that may result when using each glycaemic test.

This analysis also has limitations. NHANES includes only a single blood sample, which precludes examining confirmatory glycaemic tests that are recommended to diagnose diabetes (5). We did not use 2-hour post load glucose to define dysglycemia because it was collected for NHANES only in 2015-2016. This may have underestimated the prevalence of dysglycemia in some groups, especially among Asian Americans (29). Estimates of some participant characteristics may be statistically unreliable due to small sample sizes. Clinician diagnosis of diabetes was ascertained by participants’ self-report in NHANES, which may have underestimated the true prevalence of diagnosed diabetes. However, prior research suggests high agreement between patient and clinician reports of diabetes diagnosis (32). The ADA screening criteria are unlikely to capture many adults with type 1 diabetes, as this condition is often diagnosed before age 35 based on clinical presentation with diabetes-related symptoms or its association with other autoimmune conditions (33).

Future studies are needed to examine adoption of this screening recommendation in practice and explore its downstream impacts on glycaemic management and outcomes across all population groups. Prior research promoting adherence to dysglycemia screening guidelines have used nonrandomized designs (34, 35). Therefore, rigorous intervention studies aimed at effective and equitable implementation of the ADA screening recommendation in clinical settings are still needed. Achieving health equity in detecting dysglycemia will also require addressing social determinants of health that may hinder receipt of screening tests in some groups. Relevant factors include insurance status, educational attainment, and having a usual source of medical care, as well as others not examined here. Future research should also examine the contribution of health-related social needs to diabetes screening and glycaemic outcomes in all populations.

## 5 Conclusion

The current ADA screening recommendation for dysglycemia screening exhibits very high sensitivity and high negative predictive value across all groups defined by sex, race, and ethnicity. Our findings suggest potential for this guideline to promote health equity by maximizing detection of dysglycemia similarly across all sociodemographic groups. Observed differences in guideline performance among racial and ethnic groups were statistically significant, but generally small in magnitude and therefore unlikely to impact health equity. Given that only half of US adults report completing glucose tests previously, implementing this screening guideline effectively across all sociodemographic groups is likely to have the greatest impact on diabetes health equity.

Policymakers, clinicians, and other healthcare stakeholders can use our findings to inform implementation of preferred screening approaches. Because the ADA screening criteria exhibit very high sensitivity, following this approach will identify almost all adults with dysglycemia, which represents a significant strength. However, low specificity of the ADA criteria means that many individuals without dysglycemia would also be tested, thereby increasing screening costs across the population. While this approach may still be preferred to maximize early detection of dysglycemia, resource constraints may limit use of the ADA screening criteria in some settings. Healthcare stakeholders may also consider that following the ADA criteria will identify similar proportions of racial and ethnic groups with dysglycemia, representing another advantage of this approach.

## Data availability statement

Publicly available datasets were analyzed in this study. This data can be found here: https://wwwn.cdc.gov/nchs/nhanes/.

## Ethics statement

The studies involving humans were approved by Ethics Review Board of the National Center for Health Statistics. The studies were conducted in accordance with the local legislation and institutional requirements. The participants provided their written informed consent to participate in this study.

## Author contributions

MO’B: Conceptualization, Funding acquisition, Investigation, Methodology, Writing – original draft, Writing – review & editing. YZ: Data curation, Formal Analysis, Investigation, Methodology, Writing – review & editing. SB: Investigation, Validation, Writing – review & editing. SK: Investigation, Validation, Writing – review & editing. RA: Investigation, Validation, Writing – review & editing. MA: Investigation, Validation, Writing – review & editing. MB: Investigation, Validation, Writing – review & editing. SRB: Investigation, Validation, Writing – review & editing. GI: Investigation, Validation, Writing – review & editing. CH: Investigation, Validation, Writing – review & editing. KM: Conceptualization, Data curation, Formal Analysis, Investigation, Methodology, Resources, Supervision, Validation, Writing – review & editing.
