# Supplementary material for: Clinical performance and health equity implications of the American Diabetes Association’s 2023 screening recommendation for prediabetes and diabetes
Source: Front Endocrinol (Lausanne). 2023 Oct 13;14:1279348. doi: 10.3389/fendo.2023.1279348 (PMC10611495; doi:10.3389/fendo.2023.1279348)
Supplement: Supplementary file 1 [file Table_1.docx]

***Supplementary Material***

**Supplementary Table 1: Definitions of Current ADA Screening Criteria for Prediabetes and Diabetes**

| **Criteria** | **NHANES Definition** |
| --- | --- |
| Age ≥35 years | Self-reported age |
| Overweight or obesity (Asian Americans) | Measured BMI ≥23kg/m^2^ |
| Overweight or obesity (all other groups) | Measured BMI ≥25kg/m^2^ |
| Minority race or ethnicity | Self-reported Asian, Black, or Hispanic race or ethnicity |
| Hypertension | Measured blood pressure >130/80 mm Hg; or antihypertensive medication use |
| Dyslipidemia | Measured HDL cholesterol <35mg/dL; triglycerides >250mg/dL; or lipid-lowering medication use |
| Prediabetes | Self-reported history of prediabetes |
| Family history of diabetes | Self-reported family history of diabetes in a first-degree relative |
| History of gestational diabetes | Self-reported history of gestational diabetes or delivering a baby ≥9lb |
| History of cardiovascular disease | Self-reported history of myocardial infarction, coronary heart disease, or stroke |
| Physical inactivity | No work-, transport-, or recreational-related physical activity in a typical week |

BMI = body mass index; HDL = high density lipoprotein

**Supplementary Table 2: Characteristics of U.S. Adults with Prediabetes or Undiagnosed Diabetes by Self-Reported Sex, Race, and Ethnicity**

| **Characteristic** | **Asian** | | **Black** | | **Hispanic** | | **White** | |
| --- | --- | --- | --- | --- | --- | --- | --- | --- |
|  | **Men** | **Women** | **Men** | **Women** | **Men** | **Women** | **Men** | **Women** |
|  | **% (95% CI)** | **% (95% CI)** | **% (95% CI)** | **% (95% CI)** | **% (95% CI)** | **% (95% CI)** | **% (95% CI)** | **% (95% CI)** |
| Unweighted n | 153 | 158 | 322 | 333 | 347 | 341 | 489 | 398 |
| Mean age, years | 48.8 (46.0-51.7) | 52.5 (49.9-55.1) | 48.1 (45.7-50.5) | 50.8 (49.0-52.7) | 43.5 (41.4-45.5) | 46.7 (44.0-49.3) | 53.1 (50.4-55.7) | 58.7 (57.2-60.2) |
| Age categories, years |  |  |  |  |  |  |  |  |
| 18-34 | 20.9 (14.7-29.0) | 15.1 (9.6-23.0) | 21.2 (15.9-27.6) | 18.6 (14.5-23.5) | 27.8 (21.0-35.7) | 25.1 (18.4-33.3) | 16.8 (10.9-25.0) | 7.9 (5.3-11.7) |
| 35-44 | 19.6 (11.6-31.3) | 12.9 (8.7-18.8) | 22.8 (17.6-28.9) | 15.3 (12.3-19.0) | 28.3 (22.8-34.5) | 19.3 (14.6-25.1) | 13.3 (9.9-17.7) | 9.5 (6.4-14.0) |
| 45-70 | 52.0 (40.1-63.8) | 60.9 (54.4-67.0) | 47.5 (40.9-54.1) | 55.8 (51.5-60.1) | 39.1 (33.8-44.6) | 48.4 (41.9-54.9) | 55.5 (48.3-62.5) | 60.0 (54.1-65.7) |
| ≥71 | 7.4 (4.2-12.7) | 11.1 (7.1-16.8) | 8.6 (5.8-12.5) | 10.2 (7.0-14.6) | 4.9 (2.7-8.7) | 7.2 (4.5-11.5) | 14.3 (11.5-17.7) | 22.5 (17.9-27.9) |
| Education < high school | 12.2 (7.2-20.0) | 18.8 (12.7-26.8) | 12.5 (8.8-17.5) | 12.7 (8.8-18.0) | 43.1 (35.4-51.1) | 37.0 (30.3-44.1) | 9.0 (5.9-13.4) | 7.4 (5.1-10.6) |
| Income < Federal Poverty Level | 8.5 (4.7-15.0) | 12.2 (7.1-20.2) | 22.4 (17.0-29.0) | 29.7 (22.6-38.1) | 30.0 (23.0-37.9) | 39.4 (31.8-47.7) | 7.0 (4.3-11.0) | 7.3 (5.5-9.7) |
| Insured | 96.0 (89.8-98.5) | 93.7 (89.2-96.4) | 81.5 (73.9-87.3) | 84.7 (74.5-91.3) | 62.6 (49.4-74.1) | 71.9 (63.4-79.1) | 89.9 (85.4-93.2) | 94.4 (89.4-97.2) |
| Usual source of care | 82.9 (74.5-88.9) | 86.2 (77.9-91.7) | 78.5 (69.7-85.3) | 92.8 (87.6-95.9) | 66.5 (58.7-73.5) | 84.2 (76.8-89.6) | 83.3 (77.9-87.5) | 94.1 (90.5-96.4) |
| Weight status^a^ |  |  |  |  |  |  |  |  |
| Normal | 15.9 (11.0-22.5) | 25.5 (16.9-36.5) | 25.7 (19.2-33.5) | 8.8 (6.4-12.0) | 12.8 (9.7-16.7) | 12.1 (8.6-16.7) | 16.9 (13.4-21.0) | 19.1 (15.3-23.5) |
| Overweight | 68.3 (59.2-76.2) | 56.7 (46.3-66.5) | 31.7 (25.1-39.2) | 22.9 (17.9-28.8) | 40.0 (34.4-45.9) | 30.0 (25.0-35.7) | 38.5 (32.2-45.3) | 30.3 (24.5-36.7) |
| Obesity | 15.7 (9.9-24.0) | 17.8 (11.6-26.3) | 42.6 (37.7-47.7) | 68.3 (61.9-74.0) | 47.2 (41.7-52.8) | 57.9 (51.9-63.7) | 44.6 (37.4-52.0) | 50.6 (44.3-56.9) |
| Body mass index, kg/m^2 b^ | 26.5 (25.7-27.3) | 26.1 (25.0-27.2) | 29.5 (28.7-30.3) | 35.6 (34.5-36.7) | 30.7 (29.9-31.5) | 32.5 (31.4-33.5) | 30.2 (29.4-31.0) | 31.6 (30.5-32.7) |
| Hypertension | 56.8 (48.2-64.9) | 52.9 (43.5-62.0) | 63.7 (56.1-70.7) | 70.2 (62.1-77.2) | 43.8 (39.3-48.4) | 39.4 (33.9-45.3) | 59.7 (53.0-66.1) | 58.4 (51.9-64.5) |
| Dyslipidemia | 32.7 (24.2-42.5) | 21.1 (15.2-28.6) | 21.0 (14.5-29.2) | 23.5 (19.2-28.5) | 25.9 (21.2-31.2) | 20.4 (15.3-26.6) | 38.0 (32.6-43.7) | 35.3 (28.8-42.4) |
| Self-reported prediabetes | 21.0 (14.4-29.7) | 33.1 (22.9-45.1) | 18.5 (14.0-24.1) | 27.3 (22.8-32.3) | 19.1 (14.8-24.2) | 25.2 (19.9-31.4) | 16.8 (11.6-23.8) | 22.8 (17.1-29.6) |
| Family history of diabetes | 32.6 (24.9-41.3) | 48.4 (38.8-58.2) | 41.4 (35.7-47.3) | 56.2 (49.1-63.0) | 42.3 (34.9-50.0) | 52.0 (45.2-58.6) | 39.2 (34.5-44.0) | 46.0 (39.5-52.5) |
| History of gestational diabetes^c^ | -- | 14.2 (9.5-20.6) | -- | 13.7 (10.3-17.9) | -- | 21.1 (16.9-26.1) | -- | 19.8 (14.9-25.7) |
| History of cardiovascular disease | 4.6 (2.1-9.8) | 1.7 (0.4-6.7) | 8.2 (5.5-12.2) | 8.7 (6.3-11.9) | 4.0 (2.3-6.8) | 3.3 (1.9-5.6) | 10.3 (7.3-14.3) | 9.8 (6.9-13.7) |
| Physical inactivity | 16.5 (11.7-22.8) | 33.7 (26.5-41.7) | 21.5 (17.4-26.3) | 33.9 (26.9-41.7) | 22.3 (17.9-27.4) | 29.6 (25.0-34.6) | 11.1 (8.4-14.4) | 28.4 (23.4-34.1) |
| Received glucose test in last 3 years | 58.0 (48.0-67.4) | 60.5 (50.8-69.4) | 47.1 (39.0-55.4) | 65.5 (61.0-69.8) | 47.0 (40.7-53.4) | 58.0 (50.7-65.0) | 58.8 (53.9-63.5) | 60.5 (55.0-65.7) |
| Fasting plasma glucose, mg/dL^b^ | 115.6 (109.9-121.2) | 111.1 (107.2-115.1) | 107.9 (105.8-110.1) | 109.1 (105.8-112.4) | 115.5 (112.6-118.3) | 113.4 (109.8-117.1) | 113.0 (111.3-114.6) | 110.4 (108.3-112.6) |
| 100-125 | 69.7 (60.4-77.7) | 61.1 (51.5-69.8) | 49.8 (43.3-56.4) | 49.7 (43.6-55.9) | 80.4 (75.2-84.7) | 64.3 (59.1-69.3) | 83.4 (79.3-86.8) | 66.0 (58.3-73.0) |
| ≥126 | 9.7 (5.7-15.9) | 7.5 (3.5-15.3) | 4.3 (2.4-7.6) | 4.9 (2.9-8.1) | 7.5 (5.3-10.6) | 8.9 (5.8-13.6) | 4.4 (2.9-6.8) | 5.6 (3.3-9.3) |
| Hemoglobin A1c, % ^b^ | 5.86 (5.68-6.04) | 5.91 (5.76-6.06) | 5.90 (5.82-5.97) | 5.99 (5.88-6.09) | 5.78 (5.69-5.87) | 5.87 (5.78-5.96) | 5.59 (5.52-5.65) | 5.75 (5.70-5.81) |
| 5.7-6.4 | 57.4 (47.8-66.4) | 64.4 (55.6-72.4) | 77.0 (69.6-83.1) | 77.6 (73.8-81.0) | 48.0 (43.2-52.8) | 59.7 (53.4-65.7) | 41.5 (34.2-49.2) | 59.1 (51.1-66.7) |
| ≥6.5 | 8.1 (4.3-14.5) | 10.5 (5.9-18.0) | 6.5 (3.9-10.6) | 9.1 (6.6-12.3) | 7.7 (5.6-10.4) | 8.3 (5.4-12.5) | 1.9 (1.0-3.6) | 5.7 (3.7-8.6) |

a According to the 2023 ADA guideline, weight status was defined in Asian adults using the following BMI thresholds: Normal (18.0-22.9), Overweight (23.0-26.9), and Obesity (≥27.0). In all other racial and ethnic groups, weight status was defined using the following BMI thresholds: Normal (18.0-24.9), Overweight (25.0-29.9), and Obesity (≥30.0).

b Values are reported as mean (standard error).

c History of gestational diabetes was only assessed among those reporting female sex.

**Supplementary Table 3: Performance of ADA Screening Criteria Based on Fasting Plasma Glucose Among U.S. Adults without Diagnosed Diabetes by Self-Reported Sex, Race, and Ethnicity**

| **Population group** | **Sensitivity (95% CI)** | **Specificity (95% CI)** | **Positive Predictive Value (95% CI)** | **Negative Predictive Value (95% CI)** |
| --- | --- | --- | --- | --- |
| Total population |  |  |  |  |
| Overall | 94.6 (91.5-96.6) | 23.3 (21.0-25.8) | 40.3 (37.8-43.0) | 88.7 (82.2-93.0) |
| Asian | 97.3 (94.0-98.8) | 18.2 (13.9-23.3) | 37.6 (31.7-43.8) | 93.0 (85.6-96.7) |
| Black | 97.2 (93.2-98.9) | 19.2 (15.9-23.1) | 30.5 (26.2-35.1) | 95.0 (88.7-97.9) |
| Hispanic | 95.7 (93.0-97.4) | 18.9 (15.3-23.1) | 40.7 (37.0-44.5) | 88.3 (82.3-92.4) |
| White | 93.6 (88.5-96.5) | 25.9 (22.3-29.8) | 41.9 (38.2-45.7) | 87.6 (77.6-93.5) |
| *P*-value^a^ | 0.56 | 0.07 | <0.001 | 0.33 |
| Men |  |  |  |  |
| Overall | 92.0 (87.0-95.2) | 26.1 (22.4-30.1) | 47.4 (43.4-51.4) | 81.8 (71.5-89.0) |
| Asian | 97.3 (93.2-99.0) | 10.8 (6.6-17.1) | 40.5 (32.8-48.7) | 86.5 (71.9-94.1) |
| Black | 94.8 (85.9-98.2) | 22.6 (17.9-28.0) | 31.2 (26.2-36.7) | 92.1 (79.7-97.2) |
| Hispanic | 94.5 (90.4-96.9) | 18.5 (13.0-25.7) | 46.8 (41.0-52.6) | 81.6 (70.8-89.1) |
| White | 90.2 (82.5-94.7) | 30.6 (24.9-37.1) | 51.3 (45.2-57.3) | 79.5 (65.4-88.8) |
| *P*-value^a^ | 0.01 | 0.01 | <0.001 | 0.01 |
| Women |  |  |  |  |
| Overall | 98.0 (96.1-99.0) | 21.2 (18.6-24.1) | 34.0 (31.2-37.0) | 96.2 (92.5-98.2) |
| Asian | 97.3 (91.6-99.1) | 23.9 (17.4-31.9) | 34.6 (27.3-42.7) | 95.5 (86.5-98.6) |
| Black | 99.3 (96.9-99.8) | 16.5 (12.4-21.7) | 29.9 (24.8-35.5) | 98.4 (94.1-99.6) |
| Hispanic | 97.4 (92.8-99.1) | 19.3 (15.6-23.6) | 34.4 (29.7-39.4) | 94.4 (85.7-98.0) |
| White | 98.4 (96.6-99.3) | 22.5 (18.6-27.0) | 33.8 (29.7-38.2) | 97.3 (93.7-98.8) |
| *P*-value^a^ | 0.45 | 0.34 | 0.38 | 0.47 |

ADA = American Diabetes Association; CI = confidence interval

a *P*-values for sociodemographic differences in performance characteristics were determined using chi-square tests

**Supplementary Table 4: Performance of ADA Screening Criteria Based on Hemoglobin A1c Among U.S. Adults without Diagnosed Diabetes by Self-Reported Sex, Race, and Ethnicity**

| **Population group** | **Sensitivity (95% CI)** | **Specificity (95% CI)** | **Positive Predictive Value (95% CI)** | **Negative Predictive Value (95% CI)** |
| --- | --- | --- | --- | --- |
| Total population |  |  |  |  |
| Overall | 97.8 (96.8-98.4) | 22.5 (20.3-24.8) | 32.0 (29.7-34.4) | 96.4 (94.9-97.5) |
| Asian | 99.7 (98.1-100.0) | 18.9 (14.5-24.3) | 36.5 (31.3-42.0) | 99.4 (95.0-99.9) |
| Black | 94.6 (92.1-96.4) | 21.5 (17.5-26.2) | 46.5 (42.6-50.5) | 84.8 (79.2-89.1) |
| Hispanic | 97.2 (94.1-98.7) | 17.7 (14.7-21.1) | 31.1 (28.6-33.8) | 94.2 (88.8-97.1) |
| White | 99.0 (97.3-99.6) | 24.5 (21.2-28.1) | 29.5 (26.0-33.1) | 98.7 (96.5-99.5) |
| *P*-value^a^ | <0.001 | 0.06 | <0.001 | <0.001 |
| Men |  |  |  |  |
| Overall | 97.0 (95.4-98.1) | 23.7 (20.4-27.3) | 30.6 (27.5-33.8) | 95.8 (93.6-97.3) |
| Asian | 100.0 (. -. ) | 11.3 (7.3-17.0) | 34.3 (28.5-40.6) | 100.0 (. -. ) |
| Black | 90.2 (85.1-93.7) | 23.3 (18.2-29.1) | 45.8 (41.1-50.5) | 76.7 (67.9-83.7) |
| Hispanic | 96.7 (91.3-98.8) | 16.5 (12.3-21.9) | 30.3 (26.9-34.0) | 93.0 (84.1-97.1) |
| White | 98.9 (94.0-99.8) | 26.9 (22.1-32.4) | 27.8 (23.4-32.7) | 98.8 (93.9-99.8) |
| *P*-value^a^ | <0.001 | 0.01 | <0.001 | <0.001 |
| Women |  |  |  |  |
| Overall | 98.4 (97.0-99.1) | 21.3 (18.5-24.3) | 33.3 (29.9-36.9) | 97.0 (94.3-98.5) |
| Asian | 99.5 (96.4-99.9) | 25.8 (18.5-34.8) | 38.7 (30.2-47.9) | 99.1 (93.1-99.9) |
| Black | 98.3 (96.8-99.1) | 20.1 (15.0-26.4) | 47.1 (41.6-52.6) | 94.3 (89.2-97.0) |
| Hispanic | 97.7 (92.4-99.3) | 18.9 (15.5-22.7) | 32.0 (27.8-36.5) | 95.4 (85.3-98.7) |
| White | 99.0 (97.1-99.7) | 22.1 (18.1-26.6) | 30.9 (26.0-36.3) | 98.5 (95.3-99.5) |
| *P*-value^a^ | 0.36 | 0.18 | 0.003 | 0.17 |

ADA = American Diabetes Association; CI = confidence interval

a *P*-values for sociodemographic differences in performance characteristics were determined using chi-square tests
